# Interaction between the Hepatitis B Virus and Cellular FLIP Variants in Viral Replication and the Innate Immune System

**DOI:** 10.3390/v14020373

**Published:** 2022-02-11

**Authors:** Ah Ram Lee, Yong Kwang Park, Mehrangiz Dezhbord, Kyun-Hwan Kim

**Affiliations:** 1 Department of Precision Medicine, School of Medicine, Sungkyunkwan University, Suwon 16419, Korea; ahram2g@naver.com (A.R.L.); asal@skku.edu (M.D.); 2 Division of Chronic Viral Diseases, Center for Emerging Virus Research, National Institute of Infectious Disease, National Institute of Health, Cheongju 28159, Korea; yk1029@korea.kr

**Keywords:** hepatitis B virus, cellular FLIP (c-FLIP), viral FLIP (v-FLIP), HBx, innate immune system

## Abstract

During viral evolution and adaptation, many viruses have utilized host cellular factors and machinery as their partners. HBx, as a multifunctional viral protein encoded by the hepatitis B virus (HBV), promotes HBV replication and greatly contributes to the development of HBV-associated hepatocellular carcinoma (HCC). HBx interacts with several host factors in order to regulate HBV replication and evolve carcinogenesis. The cellular FADD-like IL-1β-converting enzyme (FLICE)-like inhibitory protein (c-FLIP) is a major factor that functions in a variety of cellular pathways and specifically in apoptosis. It has been shown that the interaction between HBx and c-FLIP determines HBV fate. In this review, we provide a comprehensive and detailed overview of the interplay between c-FLIP and HBV in various environmental circumstances. We describe strategies adapted by HBV to establish its chronic infection. We also summarize the conventional roles of c-FLIP and highlight the functional outcome of the interaction between c-FLIP and HBV or other viruses in viral replication and the innate immune system.

## 1. Introduction

Hepatitis B virus (HBV) infection remains a global health issue, affecting approximately 300 million individuals worldwide [1]. Chronic hepatitis B (CHB) leads to serious liver-related diseases, including cirrhosis and hepatocellular carcinoma (HCC).

Viruses are essentially unable to survive on their own without the help of their host. Thus, many viruses have developed distinctive strategies that utilize host cellular machinery for their survival and reproduction [2,3,4,5]. By interplaying with several host factors, viruses exploit the cellular functions for their sake. However, some interactions may conversely suppress viruses [6,7]. Viral pathogens rely on their host in nearly all steps of their life cycle such as entry, uncoating, gene expression, genome replication, exit, survival, and persistent infection.

As a result of viral encounter, the host’s innate immune cells are activated and produce interferons (IFNs) and pro-inflammatory cytokines against viral infection [8,9]. To counteract the host immune response, viruses trigger immunosuppressive pathways or adopt different strategies to escape from pathogen-sensing mechanisms [6,10,11,12]. As a typical non-cytopathic and stealth virus, HBV has evolved to evade the host immune system [13]. Therefore, HBV-induced liver damage is instead considered as the outcome of dysregulated host immune response and may not be due to the direct catastrophic effect of a virus on infected host cells [14].

Herein, we aim to recapitulate the current knowledge on the relationship between HBV and host proteins by specifically focusing on the interplay between HBV X protein (HBx) and the cellular FADD-like IL-1β-converting enzyme (FLICE)-like inhibitory protein (c-FLIP). HBx is best known to play an important role in HBV replication and the pathogenesis of HCC [15,16]. c-FLIP has also been reported to regulate several proteins involved in cell survival, proliferation, and carcinogenesis in a variety of cells through diverse signaling pathways [17,18]. These regulatory effects are often mediated by both direct interactions with cellular factors as well as indirect activation of signaling pathway components.

## 2. FLICE-like Inhibitory Proteins (FLIPs)

### 2.1. FLIP Variants

**Figure 1:**
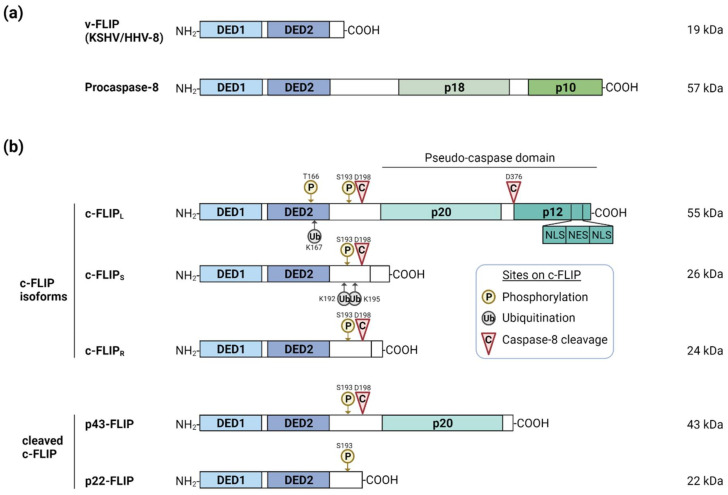
Schematic representation of procaspase-8 and FLIP variants. Structures of v-FLIP, procaspase-8 (**a**), c-FLIP isoforms and cleaved products (**b**) are depicted. All proteins commonly share DED1 and DED2 domains. Several post-translational modification sites (phosphorylation and ubiquitination) or caspase-8 cleavage sites on c-FLIP are indicated. c-FLIP: cellular FLIP; v-FLIP: viral FLIP; DED: death-effector domain; NLS: nuclear localization signal; NES: nuclear export signal. This illustration was created with BioRender.com.

Since FLIP was first found in Kaposi’s sarcoma-associated herpesvirus (KSHV), which is also referred to as human herpesvirus (HHV-8), it was named viral FLIP (v-FLIP) [19]. Tight regulation of cell death and proliferation is critical for the maintenance of cellular homeostasis in living organisms. v-FLIP is able to inhibit apoptosis and induce cell growth by interrupting the host cell death machinery [19,20]. It binds to procaspase-8, a key molecule of apoptosis initiation, and blocks its maturation, thereby inactivating the downstream apoptosis cascade [21].

c-FLIP, the homolog of v-FLIP, is mainly expressed as three isoforms: c-FLIP_L_ (long form), c-FLIP_S_ (short form), and c-FLIP_R_ (Raji form) (Figure 1). c-FLIP_L_ is cleaved by caspase-8, generating two N-terminal fragments (p43-FLIP and p22-FLIP). All three c-FLIP isoforms share structural homologies with caspase-8, as c-FLIP isoforms and caspase-8 both contain N-terminal tandem death-effector domains (DEDs), allowing their recruitment to the death-inducing signaling complex (DISC). In addition, c-FLIP_L_ contains a catalytically inactive pseudo-caspase domain, which consists of a large (p20) and a small (p12) domain that shares the most homology with caspase-8. The pseudo-caspase domain contains a nuclear localization signal (NLS) and nuclear export signal (NES), which enable shuttling between the nucleus and the cytoplasm. The subcellular localization of c-FLIP has been shown to correlate with functional and pathological outcomes such as patient survival rate and malignant progression in diverse diseases [22,23,24]. Other isoforms lack the pseudo-caspase domain and their C-terminus differ from each other.

### 2.2. Cellular Functions of FLIP Variants

c-FLIP is known to have multiple functions in various signaling pathways that regulate cell fate. In death receptor-mediated apoptosis signaling, c-FLIP is a master anti-apoptotic modulator. c-FLIP_S_ and c-FLIP_R_ isoforms have been observed to block procaspase-8 activation and apoptosis [25,26]. In a similar way, c-FLIP_L_, which has a structural similarity with caspase-8, plays a central role in interfering with extrinsic apoptotic signaling by binding to FAS-associated death domain (FADD), caspase-8 or caspase-10, and tumor necrosis factor-related apoptosis-inducing ligand (TRAIL) receptor 5 (DR5), forming an apoptotic inhibitory complex (AIC) [27,28]. Thus, the interaction between c-FLIP and apoptosis-associated mediators inhibits subsequent activation of the caspase cascade, resulting in the prevention of cell death.

Furthermore, c-FLIP has been shown to stimulate cytoprotection and proliferation through activation of extracellular signal-regulated kinase (ERK) and nuclear factor kappa-light-chain-enhancer of activated B cells (NF-κB) signaling pathways [29,30]. In addition to c-FLIP_L_, p43-FLIP was also reported to have a role in the activation of the ERK and NF-κB pathways [31,32]. c-FLIP is similarly important for protecting T lymphocytes from apoptosis [33] by promoting the survival of immunosuppressive immune cells (e.g., myeloid-derived suppressor cells, MDSCs) and maintaining regulatory T cells (T_regs_) [34,35]. In addition, c-FLIP_R_ transgenic mice infected with bacteria showed less liver necrosis and better bacterial clearance compared to wild-type mice, indicating that c-FLIP_R_ expression supports an efficient T cell activation [36]. Conversely, c-FLIP_S_ inhibits activation of caspase-8 in T cells, resulting in the reduced activity of NF-κB and T cell survival [37].

c-FLIP has also been reported to play a key role in regulating another type of cell death called ‘necroptosis (necrosis)’ as well as modulating ‘autophagy’ as an essential cellular process [18,38,39]. Necroptosis was understood as passive cell death induced under extreme conditions, but recently, many reports have demonstrated that necroptosis is also programmed and regulated by intracellular molecules. This form of cell death is caspase independent and utilizes the receptor-interacting protein (RIP)1 and RIP3 kinases when caspases are inhibited [40]. Ripoptosome, which is the necrotic signaling platform, is composed of RIP-1, FADD, and caspase-8 [40]. c-FLIP_L_, a caspase-8 paralogue, is able to intervene with the formation of this complex. However, depending on the expression level or type of c-FLIP isoforms, different effects on the necrotic complex have been observed. For instance, unlike the c-FLIP_L_ that negatively regulates the necroptosis, c-FLIP_S_ promotes RIP3-mediated necroptosis [38]. Therefore, c-FLIP isoforms determine whether cell death occurs by caspase-dependent apoptosis or through the RIP3-mediated necroptosis. In addition, c-FLIP or v-FLIP suppresses autophagy by prohibiting autophagy-related 3 (Atg3) from binding and processing the microtubule-associated protein 1 light chain 3 (LC3), which is an essential component for autophagosome formation [39].

Dysregulation of c-FLIP is involved in several diseases, including some types of cancer [24,41,42,43,44,45], Alzheimer’s disease [46], and chronic obstructive pulmonary disease (COPD) [47]. Notably, the elevated expression of c-FLIP is highly associated with cancer malignancy, poor prognosis, and resistance to chemotherapy [24,41,44,45,48,49,50]. Silencing of c-FLIP sensitizes cancer cells to death signals and chemotherapeutic agents, implying that c-FLIP can be considered as a promising therapeutic target for cancer [51,52]. Modulation of c-FLIP by TNF-α/NF-κB axis was also suggested as a strategy to treat mutant melanomas [50].

Collectively, c-FLIP functions as a central mediator in the complex interplay between apoptosis, autophagy, and necroptosis, which are essential processes for maintaining cellular homeostasis.

## 3. HBV Life Cycle

HBV is a small, enveloped virus with a partially double-stranded, relaxed circular (RC) DNA genome of approximately 3.2 kb. HBV enters hepatocytes via interaction with host membrane proteins, heparan sulfate proteoglycan (HSPG) [53,54,55], and its specific receptor, sodium taurocholate co-transporting polypeptide (NTCP/SLC10A1) [56]. Recently, epidermal growth factor receptor (EGFR) has also been identified as a host cofactor in the internalization of HBV-NTCP [57]. The binding of HBV to its receptor on the cell surface facilitates virus internalization via receptor-mediated endocytosis [58,59,60]. The incoming nucleocapsids are disassembled and the HBV genome is delivered into the nucleus, where it is repaired to form covalently closed circular DNA (cccDNA), the viral persistence reservoir [61]. cccDNA serves as a template for transcription of all viral mRNAs that are translated into four proteins: polymerase, core, surface, and HBx. Various cellular factors are involved in the conversion of relaxed circular DNA (rcDNA) into cccDNA, including the removal of polymerase, RNA redundancy, and RNA primer, completion of viral (+) strand, and ligation of DNA ends. Lei Wei et al. have recently reported that five core components of DNA lagging-strand synthesis are essential for cccDNA formation: proliferating cell nuclear antigen (PCNA), the replication factor C (RFC) complex, DNA polymerase delta (POLδ), flap endonuclease 1 (FEN-1), and DNA ligase 1 (LIG1) [62]. Other host-derived factors related to DNA repair machinery had also been presumably considered as cofactors for cccDNA establishment [63,64,65,66,67,68]. Furthermore, cccDNA stably exists in the nucleus of HBV-infected cells in the form of minichromosome and is associated with cellular histones as well as host and viral proteins, which maintain its organization and regulate the epigenetic modification of cccDNA [69,70,71]. Among viral proteins, HBx and core are associated with cccDNA and have an essential role in cccDNA transcription [70,72]. Various cellular transcription factors are recruited to viral promoter/enhancer regions of cccDNA to control its transcriptional activity [73,74,75].

These findings indicate that HBV takes advantage of host cellular factors in cccDNA biosynthesis, maintenance, and activation.

## 4. HBV and Innate Immune System

In the early stages of viral infection, host cells recognize the viral components through pattern-recognition receptors (PRRs) and activate the innate immune system [9]. In response to HBV infection, hepatocytes and hepatic non-parenchymal cells such as liver sinusoidal endothelial cells (LSECs) and kupffer cells, along with HBV-specific T cells and B cells sense HBV components [76]. Previous studies have reported that HBV pgRNA and DNA, which are exposed during viral replication, are recognized by retinoic acid-inducible gene I (RIG-I) and cyclic GMP-AMP synthase (cGAS)-stimulator of interferon (IFN) genes (STING) signaling pathway, respectively [77,78]. More specifically, a report has shown that the naked HBV genome is sensed by cGAS but the encapsidated genome can evade viral sensing by the innate immune system [79]. In addition, toll-like receptors (TLRs) as well as melanoma differentiation-associated protein 5 (MDA5) are functionally expressed in HepaRG cells [80]. These cytosolic PRRs activate certain adaptor molecules such as TANK-binding kinase 1 (TBK1), IFN regulatory factors (IRFs), and MyD88, which subsequently leads to the induction of IFNs and pro-inflammatory cytokines [9].

However, in the late stages of infection, HBV dysregulates the host immune function as a contradictory strategy to escape from viral recognition which leads to the establishment of viral persistence [81].

### 4.1. Interferons

During acute HBV infection, it is well known that type I/II and III IFNs are induced in the liver. Type I IFN (IFN-α/β) exerts the antiviral activity in different steps of the HBV life cycle. Previously, it was reported that the unidentified soluble factors released by IFN-α-treated HepaRG cells restrict HBV entry by competing with binding to HSPG [82]. In addition, IFN-α/β inhibit HBV replication by destabilization of the pgRNA-containing nucleocapsids in transgenic mice and murine hepatocytes [83,84]. Moreover, IFN-α suppresses HBV replication at the transcriptional level by epigenetic modification of cccDNA. Mechanistically, IFN-α attenuates the binding of signal transducer and activator of transcription (STAT) 1 and STAT2 to cccDNA, and also induces hypo-acetylation of H3K9 and H3K27, which further repress recruitment of histone deacetylase (HDAC) 1 to cccDNA [85,86]. These epigenetic modifications regulate the stability of cccDNA and eventually decrease the transcription of HBV RNA. Importantly, IFN-α and lymphotoxin-β are capable of inducing deamination of cccDNA via APOBEC3A and APOBEC3B, respectively, leading to its degradation by base excision repair enzymes [87]. Lastly, it has been indicated that the IFN-α-inducible tetherin, a host restrict factor of virus egress, inhibits HBV release. Tetherin physically interacts with HBV’s large surface protein and entraps the HBV virion in the intracellular multivesicular body [88].

IFN-γ, as another major promoter of innate immunity and inflammatory responses, is mainly produced by hepatic immune cells during HBV infection and functions in harmony with other antiviral cytokines. IFN-γ and TNF-α, produced by cytotoxic T cells, reduce intracellular HBV DNA and RNA in HBV transgenic mice via non-cytopathic mechanism [89]. This additionally induces the destabilization of cccDNA by APOBEC3A or APOBEC3B and consequently reduces the accumulation of cccDNA in primary human hepatocytes (PHHs) and HepaRG cells [90]. Reportedly, similar to type I IFN, IFN-γ reduces HBV replication by inhibiting the formation and/or accelerating decay of replication-competent nucleocapsid [83,84]. Of note, the inhibitory effect of type I IFNs requires proteasome activity as it has been shown that blocking proteasomal degradation pathway could limit the IFN function [91]. Finally, IFN-γ produced by hepatic natural killer (NK) cells solely participates in DEAD box polypeptide 60 (DDX60)-mediated degradation of cytoplasmic HBV RNA [92].

Regarding type III IFN, it has been revealed that the IFN-λ is induced by RIG-I pathway following recognition of the HBV pgRNA [77]. Similar to type I and II IFN, IFN-λ exhibits anti-HBV activity through the induction of nucleocapsid dysfunction [93]. Furthermore, IFN-β, IFN-λ1, and IFN-λ2 induce deamination and degradation of cccDNA by APOBEC3A [94]. Interestingly, the core-binding factor beta (CBFβ) induced by IFN-λ inhibits HBV replication through interaction with HBx [95], which leads to the interruption of HBx-DDB1-Structural maintenance of chromosome 5/6 (SMC5/6) complex, which is important for HBx-mediated cccDNA transcription [96].

Collectively, IFNs directly control HBV replication by regulation of viral parameters in different steps of the HBV life cycle. The indirect antiviral effect of IFNs via Janus tyrosine kinase (JAK)/STAT-interferon stimulated genes (ISG) signaling pathway is summarized in the following Section 4.3.

### 4.2. Pro-Inflammatory Cytokines

Former studies have stated that the pro-inflammatory cytokines control HBV in the early steps of its life cycle. Interleukin-6 (IL-6) blocks HBV entry by down-regulation of NTCP expression, which results in the inhibition of cccDNA formation. This further suppresses HBV gene expression and transcription through the reduction of the hepatocyte nuclear factor (HNF) 1α and HNF4α expression levels by the MAPK family [97,98]. Transforming growth factor (TGF) β1 accelerates cccDNA deamination and degradation through activation-induced cytidine deaminase (AID) and inhibits HBV replication by HNF4α suppression and attenuating core promoter activity [99,100]. Similarly, IL-4 weakens the activity of both surface promoter II (preS2) and core promoter through decreasing the level of CAAT-enhancer-binding protein (C/EBP) α [101]. Furthermore, TNF-α- and IFN-γ-inducible p22-FLIP, hepatocystin, and IL-32 suppress HBV transcription by dysregulating HNF3β and HNF4α via ERK1/2 activation in HepG2 and PHHs [102,103,104]. Among IFNs and cytokines, IL-1β exhibits the strongest inhibitory effect on HBV DNA and RNA in HepaRG cells and PHHs [105]. It has been suggested that the monocyte chemotactic protein-induced protein 1 (MCPIP1) is involved in the HBV inhibitory effect of IL-1β [106].

Collectively, these studies indicate that the pro-inflammatory cytokines that are secreted by hepatic immune cells (mainly kupffer cells) upon viral encounter [98] may be responsible for the prevention of HBV propagation in hepatocytes prior to the IFN production.

### 4.3. Interferon Stimulated Genes (ISGs)

IFN-JAK/STAT signaling pathway induces hundreds of IFN-stimulated genes (ISGs), which participate in the control of the HBV life cycle [107]. Myxovirus resistance protein A (MxA), 2′-5′-oligoadenylate synthetase (OAS) and RNA-activated protein kinase (PKR) induced by type I IFN are the most well-known ISGs. MxA interferes with capsid assembly and pgRNA encapsidation through interaction with core protein [108]. 2′-5′-OAS binds to the ribonuclease L (RNase L), resulting in the decay of HBV RNAs [109]. PKR increases the eukaryotic initiation factor 2α (elF2α) phosphorylation and down-regulates intracellular capsid but not pgRNA levels, suggesting that PRK inhibits HBV replication at the translational level [110]. ISG20 induced by type I IFN also suppresses HBV replication by directly binding to the epsilon stem-loop structure of pgRNA and core promoter region [111,112]. A recent study identified that ISG20 is responsible for APOBEC-mediated cccDNA degradation [113]. Several studies have demonstrated that the tripartite motif (TRIM) proteins are part of ISGs and are capable of working as transcriptional repressors; for example, TRIM22, which is induced by IFN-α, inhibits HBV replication by reducing viral transcription by binding to the HBV core promoter region [114]. According to our previous study, to conquer host immunity, HBx represses IFN-α or IFN-γ-induced transcription of TRIM22 via a single CpG methylation in its 5′ untranslated region (UTR) [115]. Likewise, several other TRIM proteins, including TRIM41, have been shown to inhibit HBV enhancer and core promoter activity [116]. Recently, it has been observed that TRIM21 inhibits HBV replication via ubiquitination of HBV polymerase [117] or HBx [118] through its E3 ubiquitin ligase activity.

Taken together, type I IFNs both directly and indirectly regulate host proteins that participate in anti-viral activities in multiple steps of the HBV life cycle.

## 5. Interaction between Virus and FLIP Variants

### 5.1. HBV

#### 5.1.1. Apoptosis

**Figure 2:**
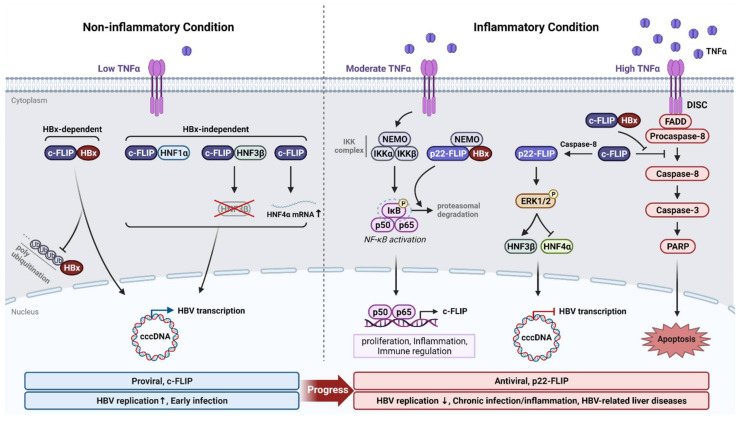
Proviral and antiviral roles of FLIP variants and corresponding signaling pathways induced in the presence or absence of inflammatory cytokine. Prior to induction of host innate immunity, c-FLIP enhances HBV transcription and replication by protecting HBx from proteasomal degradation. Additionally, c-FLIP up-regulates HNF1α and HNF4α levels that are essential co-factors for HBV genome expression. The inhibitory transcription factor HNF3β is degraded to facilitate HBV RNA production (**left**). Following the moderate induction of TNF-α, HBx-p22-FLIP-NEMO ternary complex is formed that further enhances canonical NF-κB pathway by proteasomal degradation of phosphorylated IκB, which amplifies c-FLIP transcription by p50 and p65. This renders antiviral activity of c-FLIP. When TNF-α is highly secreted, c-FLIP is cleaved by caspase-8 to form p22-FLIP which consequently phosphorylates ERK1/2. Activated ERK1/2 accelerates and blocks HNF3β and HNF4α, respectively which impedes HBV transcription from cccDNA. Lastly, high level of TNF-α activates apoptosis by direct activation of cascades. This event is accelerated following the interaction between c-FLIP and HBx (**right**). HNF: hepatocyte nuclear factor; TNF-α: tumor necrosis factor-α; NF-κB: nuclear factor kappa-light-chain-enhancer of activated B cells; IκB: I-kappa-B; ERK1/2: extracellular signal-regulated kinase 1/2; NEMO: NF-kappa-B essential modulator. This illustration was created with BioRender.com.

HBx is a multifunctional regulator that is involved in signaling pathways, transcriptional activation, epigenetic modification, cell growth, pro/anti-apoptosis, and progression of cancer, which strongly indicates its implication in the pathogenesis of HBV-related diseases. Regarding pro-apoptotic function, extensive studies have suggested that HBx expression in hepatocytes is associated with apoptotic pathways [119,120,121]. In line with these results, we previously reported that the pro-apoptotic function of HBx is mediated through interaction with c-FLIP variants [122] (Figure 2). Upon TNF-α activation, HBx forms a complex with c-FLIP_L_ or c-FLIP_S_ and abrogates its recruitment to DISC, thereby enhancing the activation of the apoptosis pathway [122]. Although c-FLIP generally protects hepatocytes from death-inducing signals, HBx renders infected hepatocytes susceptible to apoptotic stimuli, suggesting that intervention of HBx/c-FLIP may be a therapeutic target for HBV-associated diseases.

Due to the various experimental conditions and model systems, different effects of HBx on apoptosis have been reported [123,124]. A report showed that HBx, stably expressed in Hep3B cells, inhibited TGF-β-induced apoptosis via the activation of PI3K [123]. In PHHs, mouse erythroleukemia cell line (DP-16), and mouse embryo fibroblasts, HBx protected the cells against Fas-mediated apoptosis through upregulation of stress-activated protein kinase/c-JUN N-terminal kinase (SAPK/JNK) pathway [124].

#### 5.1.2. Antiviral Factor

Among the FLIP variants and cleavage forms, only p22-FLIP has been shown to exert anti-HBV effect. p22-FLIP is involved in TNF-α-mediated inhibition of HBV replication [102] (Figure 2). Particularly, p22-FLIP is cleaved by procaspase-8 from c-FLIP_L_ or c-FLIP_S_ following induction of the TNF-α/NF-κB signaling pathway. Moreover, p22-FLIP strongly impedes HBV DNA, RNA, and protein levels by diminishing the activity of viral enhancers. Mechanistic studies have also revealed that p22-FLIP enhances HNF3β expression and conversely reduces HNF4α expression through the activation of ERK1/2, which eventually results in the suppression of HBV at the transcriptional level. We have previously revealed that the N-terminus of DED1 domain (helixes α1–α3) of p22-FLIP is responsible for the p22-FLIP-mediated inhibition of HBV replication. Interestingly, the expression of endogenous p22-FLIP is relatively more abundant in PHHs than in cancer cells, implying a possible role of p22-FLIP as a host restriction factor in inflammatory condition [102]. 

According to the evidence stated above and given that no histological damage was observed in p22-FLIP-overexpressed mouse liver, we identified p22-FLIP as a novel antiviral molecule involved in noncytopathic viral clearance.

#### 5.1.3. Proviral Factor

As mentioned earlier, HBx modulates the activity of numerous enzymes and components involved in intracellular signaling pathways. Of note, HBx role as a transactivator on the cellular and viral promoters and enhancers was frequently observed [125,126]. HBx acts as an essential factor in promoting HBV replication [127]. Reportedly, HBx deficiency had a minor impact on HBV transcription and replication in Huh7 cells; nevertheless, its absence significantly impaired HBV replication in HepG2 cells, implying that HBx function may depend on unknown host cell-specific factors [127]. Moreover, several studies have demonstrated mechanisms by which HBx regulates host proteins to enhance viral replication [16,128,129,130,131]. Recent reports have revealed that HBx induces degradation of SMC5/6 in order to enhance HBV replication [96,132].

Based on the finding that HBx and c-FLIP interacts and the fact that protein–protein interactions generally regulate mutual stability, we previously investigated the effect of HBx/c-FLIP interaction on HBV replication [133] (Figure 2). Our previous report showed that HBx is stabilized by c-FLIP_L_ or c-FLIPs, where DED1/2 are associated with binding to HBx and DED1 is required for HBx stabilization, thus protecting it from proteasome-mediated degradation and contributing to robust HBV transcription and replication [133]. Furthermore, in an HBx-independent manner, c-FLIP regulates HNFs, which are crucial for HBV replication as well as hepatocyte differentiation [133].

Collectively, c-FLIP could serve as either proviral or antiviral factors, depending on the cellular milieu, such as concentration of TNF-α, implying that the ratio of c-FLIP to p22-FLIP may determine the fate of HBV. Equally important, the interaction of c-FLIP variants with HBx may result in different physiological outcomes. These results highlight the multifunctional role of c-FLIP on HBV propagation in the presence or absence of HBx.

#### 5.1.4. Cell Proliferation

Among c-FLIP variants, c-FLIP_L_ or c-FLIP_S_ can be cleaved to p22-FLIP by TNF-α stimuli, and activate NF-κB via its interaction with the IkB kinase (IKK) complex, which consists of IKK-α, IKK-β, and NEMO (NF-κB essential modulator also known as IKK-γ) [134] (Figure 2). Another study showed that all c-FLIP isoforms participate in the activation of the IKK complex by different mechanisms [135]. NF-κB is a well-known transcription factor involved in immune response, inflammation, cell survival, and proliferation [136]. There have been many reports that HBx activates NF-κB signaling [137,138,139]. Several reports further suggested the association of host partners in HBx-mediated NF-κB activation [140,141]. Furthermore, HBx-induced NF-κB activation has been shown to have a strong correlation with the pathogenesis of chronic inflammation and HCC.

We have previously revealed that the p22-FLIP synergistically enhances HBx-induced NF-κB activation by forming a ternary complex composed of HBx-p22-FLIP-NEMO [142]. In patients chronically infected with HBV, long-term exposure to TNF-α may lead to p22-FLIP accumulation and HBx-enhanced NF-κB activation, thus placing hepatocytes in a persistent inflammatory condition. Simultaneously, NF-κB activation enhances the survival and proliferation of hepatocytes, allowing them to evade the host immune response and maintain persistent infection [143]. These perspectives may provide a clue to the mechanism by which p22-FLIP contributes to the development of HCC during chronic HBV infection. Of note, human hepatocyte division triggered by NF-κB activation may contribute to cccDNA dilution without cytolysis [144].

### 5.2. Other Viruses

Several viruses such as herpesviruses and poxviruses encode v-FLIP, which resembles c-FLIP_S_. v-FLIP is able to block extrinsic apoptosis through binding to procaspase-8 and interfering with its maturation and activation, proving that v-FLIP acts as a viral inhibitor of caspase-8 [19,21]. However, an interesting report showed that HHV-8-encoding v-FLIP reduced the expression of c-FLIP but alleviated apoptosis induced by loss of c-FLIP in intestinal epithelial cells (IECs) in a mouse model [145]. HHV-8-derived v-FLIP has also been reported to potentiate NF-κB signaling by direct interaction with the IKK complex [146,147]. Furthermore, MC159 and MC160, v-FLIP from molluscum contagiosum virus (MCV), which belongs to the poxviridae family, inhibits the activation of interferon regulatory factor (IRF3) by a different mechanism [148]. Noteworthy, the interaction of MC159 and Fas/FADD disrupts FADD self-association, consequently leading to inhibition of caspase activation in DISC [149,150]. Unlike v-FLIP of HHV, the anti-apoptotic function of MC159 is not exerted by the modulation of NF-κB activity. Rather, MC159 hijacks SH3BP4, a host factor involved in autophagy regulation, and suppresses autophagy, which enables MCV to evade antiviral host immunity and establish persistent infection [151]. However, another study showed that MC159 enhances innate immunity by promoting NF-κB induction in a MC159-transgenic mouse model infected with vaccinia virus (VV) [152].

In addition to HBV, associations with other viral proteins and c-FLIP have been frequently observed. Hepatitis C virus (HCV) core protein sustains the expression of c-FLIP to block TNF-α-induced apoptosis [153]. In comparison with this, one study showed that HCV core protein sensitizes cells to TNF-α-induced apoptosis by binding to FADD and facilitating recruitment to TNF receptor 1 (TNFR1), demonstrating that the effects of HCV core protein may vary depending on different cell origins [154]. In line with this observation, another study revealed that HCV core, nonstructural protein (NS) 4B, and NS5B enhances TNF-α-mediated cell death via NF-κB inactivation, following reduction of NF-κB-dependent anti-apoptotic proteins, such as B-cell lymphoma-extra large (Bcl-xL), (X-linked inhibitor of apoptosis (XIAP), and c-FLIP [155]. Meanwhile, HCV NS5A protected human hepatoma cells from lipopolysaccharide (LPS)-induced apoptosis by increasing the expression levels of Bcl-2 and c-FLIP [156].

Herpes simplex virus type-1 (HSV-1) is one of the most common viruses that infect humans. HSV-1 induces proteasome-dependent degradation of c-FLIP in immature dendritic cells (iDCs), thereby culminating in cell death and weakening antiviral immune response [157]. In addition, HSV-1 encodes latency-associated transcript (LAT) sequences, which is important for viral latency and reactivation, and was identified as a substitute for c-FLIP [157]. 

c-FLIP was reported to inhibit human immunodeficiency virus-1 (HIV-1) replication in jurkat cells, CD4+ T cells, and peripheral blood mononuclear cells (PBMCs) by two distinct mechanisms [158]. c-FLIP magnified the expression of viral restriction factors while attenuating HIV-1-induced signaling pathways essential for its survival [158]. c-FLIP-mediated inactivation of FADD also inhibited HIV-1 replication [158]. Moreover, several studies have demonstrated that HIV infection regulates the cellular apoptotic pathway. For instance, TRAIL-mediated apoptosis was enhanced in HIV-1-infected monocyte-derived macrophages (MDMs) by down-regulating the expression of TRAIL decoy receptors and c-FLIP [159]. On the other hand, HIV-infected dendritic cells (DCs) can escape from NK cells-induced TRAIL killing by the up-regulation of c-FLIP and cellular inhibitor of apoptosis 2 (c-IAP2) [160]. Similar observation reported that HIV-1 Tat protein down-regulated caspase-10 while simultaneously up-regulating c-FLIP, thus rendering cells resistant to death-inducing signals [161]. HIV-1 Tat protein also protected CD4+ T lymphocytes from FasL-mediated apoptosis by enhancing the expression of NF-κB-dependent anti-apoptotic proteins, including Bcl-2, c-FLIP, XIAP, and c-IAP2 [162].

Human cytomegalovirus (HCMV) is also a prevalent pathogen in humans. HCMV viral immediate early 2 (IE2) protein promotes the expression of c-FLIP to protect HCMV-infected human retinal cells from apoptosis and concomitantly allows HCMV to avoid Fas-mediated killing by T lymphocytes [163]. Furthermore, HCMV-encoded chemokine receptor US28 induces apoptosis, which is neutralized by c-FLIP and HCVM antiapoptotic protein IE1 [164]. HCMV-induced delayed cell death is also mediated by significant elevation of c-FLIP and reduced pro-apoptotic proteins [165].

Moreover, IL-24 excludes c-FLIP from TLR3-associated signaling complex facilitated by influenza A virus (IAV), converting it into a death-inducing signaling complex (TLR3 DISC) that leads to apoptosis [166]. In c-FLIP_L_-transgenic mice infected with coxsackievirus B3 (CVB3), c-FLIP_L_ expression in T cells augments cell survival pathways and T-cell receptor (TCR) signaling, thus lowering the severity of CVB3-induced myocarditis [167]. In contrast to this finding, c-FLIP_S_-transgenic mice were vulnerable to CVB3 infection, indicating that c-FLIP_L_ and c-FLIP_S_ exhibit opposite effects [168].

In summary, viral proteins modulate c-FLIP by various mechanisms in order to tackle the host innate defense system and v-FLIP itself exerts cytoprotective function for virus survival and persistence (Table 1).

## 6. Conclusions

During evolution, viruses have developed distinct strategies in order to readily exploit the host machinery and facilitate their propagation. This is accomplished by direct or indirect interaction with numerous cellular factors. Virus–host interaction is a double-edged sword, with either exploitive or antagonistic consequences. Therefore, different cellular responses may occur depending on complex virus–host interplay.

HBV relies on diverse host machinery to establish its genome, and concomitantly regulates cellular factors to promote its replication, evade the host defense system, and ultimately achieve persistent infection. c-FLIP, as a multifunctional protein, is engaged in many cellular pathways that determine cell fate. Thus, its expression is tightly controlled by sophisticated regulatory mechanisms and is closely coordinated with other signaling pathways. Dysregulated c-FLIP was proven to be correlated with various diseases as well as viral pathogenesis. Our comprehensive evaluation of the HBV–c-FLIP relationship and the studies reviewed above expanded our knowledge on how HBV hijacks and subverts cellular functions for its advantage.

The most challenging goal will be to understand the contradictory crosstalk between HBV or other viruses and major cellular components such as c-FLIP in a variety of biological conditions in vitro and/or in vivo. The definite molecular mechanisms underlying the association between cellular factors and viruses require further clarifications. Understanding virus–host interplay will shed light on discovery of druggable targets.

## Figures and Tables

**Table 1:** Effect of FLIP variants on host and viruses.

	Virus	Viral Partner	FLIP Isoform	Effect on Virus	Function	Reference
viral FLIP	HHV-8(KSHV)	v-FLIP		Proviral	Inhibition of apoptosis by binding to caspase-8	[19,21,145]
		v-FLIP		Antiviral	Activation of NF-κB via interaction with IKK complex	[146,147]
	MCV	v-FLIP (MC159, MC160)		Proviral	Inactivation of IRF3 by different mechanisms	[148]
		v-FLIP (MC159)		Proviral	Inhibition of apoptosis via interaction with Fas/FADD	[149,150]
		v-FLIP (MC159)		Proviral	Suppression of autophagy by interacting with SH3BP4	[151]
		v-FLIP (MC159)		Antiviral	Activation of NF-κB in the presence of Vaccinia virus	[152]
cellular FLIP	HBV	HBx	c-FLIP_L/S_	Antiviral	Enhancement of pro-apoptotic function of HBx	[122]
		HBx	p22-FLIP	Tumorigenesis	Activation of NF-κB by forming a ternary complex (HBx-p22-FLIP-NEMO)	[142]
		-	p22-FLIP	Antiviral	Activation of ERK1/2 and regulation of HNFs	[102]
		HBx	c-FLIP_L/S_	Proviral	HBx stabilization and regulation of HNFs	[133]
	HCV	Core	c-FLIP_L/S_	Proviral	c-FLIP stabilization and blocking TNF-α-induced apoptosis	[153]
		Core, NS4B and NS5B	c-FLIP_L/S_	Antiviral	Enhancement of TNF-α-mediated cell death via NF-kB inactivation	[155]
		NA5A	c-FLIP_L/S_	Proviral	increasing the expression levels of Bcl-2 and c-FLIP to protect cells from LPS-induced apoptosis	[156]
	HSV-1		c-FLIP_L/S_	Proviral	Proteasome-dependent degradation of c-FLIP in iDCs	[157]
	HIV-1	-	c-FLIP_L/S_	Antiviral	① Enhancing the expression levels of host restriction factors and inactivating HIV-1-induced signaling pathway ② Inactivation of FADD	[158]
		-	c-FLIP_L/S_	Antiviral	Down-regulating the expression of TRAIL decoy receptors and c-FLIP in MDMs	[159]
		-	c-FLIP_L/S_	Proviral	accelerating the expression levels of c-FLIP and c-IAP2 in DCs in order to escape from NK cell-induced TRAIL-mediated apoptosis	[160]
		Tat	c-FLIP_L/S_	Proviral	Increase the expression levels of c-FLIP and decrease caspase-10	[161]
		Tat	c-FLIP_L/S_	Proviral	Inhibition of FasL-mediated apoptosis by NF-κB activation	[162]
	HCMV	IE2	c-FLIP_L/S_	Proviral	Increasing the expression level of c-FLIP to avoid Fas-mediated apoptosis by T cells	[163]
		US28	c-FLIP_L/S_	Proviral	Attenuation of apoptotic function of US28 by c-FLIP and IE1	[164]
		-	c-FLIP_L/S_	Proviral	Delaying cell death by increasing level of c-FLIP and decreasing the level of pro-apoptotic proteins	[165]
	IAV	-	c-FLIP_L/S_	Antiviral	Conversion of c-FLIP/TLR3-mediated signaling complex to atypical TLR3-associated DISC	[166]
	CVB3	-	c-FLIP_L_	Antiviral	Enhancement of T cell survival pathways and TCR signaling	[167]
		-	c-FLIP_S_	Proviral	Reduction of the mitochondrial antiviral signaling protein (MAVS), escalating caspase-8 activity and type I IFN production	[168]

HHV-8 (KSHV): Human herpesvirus 8 (Kaposi’s sarcoma-associated herpesvirus); MCV: Molluscum contagiosum virus; HBV: Hepatitis B virus; HCV: Hepatitis C virus; HSV-1: Herpes simplex virus type-1; HIV-1: Human immunodeficiency virus 1; HCMV: Human cytomegalovirus; IAV: Influenza A virus; CVB3: Coxsackievirus B3.
